# Mental health prevention in Poland and selected European Union member states: a comparative analysis of systemic interventions

**DOI:** 10.3389/fpubh.2026.1719796

**Published:** 2026-04-24

**Authors:** Dorota Weber, Anna Pacian, Monika Baryła-Matejczuk, Teresa Kulik, Anna Ślifirczyk, Beata Strzelecka

**Affiliations:** 1 Institute of Psychology and Human Sciences, WSEI University, Projektowa, Lublin, Poland; 2 Department of Health Education at the Department of Nursing Development, Faculty of Health Sciences, Medical University in Lublin, Lublin, Poland; 3 Radom Higher School, Radom, Poland

**Keywords:** European Union, health policy, mental health, prevention, systematic interventions

## Abstract

**Background:**

Mental health disorders affect approximately 84 million people in the European Union (18.4% of the total EU population), with significant variations in preventive care approaches across member states. Despite growing policy attention, significant implementation gaps persist, particularly in Central and Eastern European countries. This study examines mental health prevention strategies in Poland in comparison with selected EU member states.

**Methods:**

A comprehensive document analysis was conducted examining strategic documents, legal acts, and reports from 2013–2025. We analyzed national health programs, legal regulations, and reports from international organizations (WHO, EU) across Poland and ten purposively selected EU member states. Search strategy covered policy databases (WHO, European Commission, national government websites, OECD, Eurostat), academic literature (PubMed, Web of Science, and Scopus), and grey literature. Total documents reviewed: 127 policy documents, 89 peer-reviewed articles, 34 statistical reports. Final inclusion: 42 policy documents, 28 peer-reviewed articles, 12 statistical reports.

**Results:**

Significant differences were identified in mental health prevention approaches between Poland and other EU countries. In Poland, 25% of individuals with mental health conditions receive adequate care, with 8.9 psychiatrists and 28.2 nurses per 100,000 population compared to 11.5 psychiatrists and 31.6 nurses per 100,000 in Czech Republic. Nordic countries demonstrate the highest level of comprehensive preventive programs. Universal prevention programs exist in Denmark, Finland, Netherlands, Lithuania, Latvia, Portugal, Luxembourg, and Sweden, while Poland lacks systematic solutions despite policy support.

**Conclusion:**

Systematic implementation of evidence-based mental health interventions is necessary in Poland, including early intervention, intersectoral cooperation, and targeted mental health programs for children and adolescents aged 7–18 years. The study highlights the need for comprehensive policy integration and increased resource allocation to achieve parity with leading EU member states.

## 1 Introduction

Mental health prevention represents a critical public health imperative with profound implications for individual wellbeing, societal functioning, and economic productivity. The theoretical foundations for mental health prevention derive from multiple conceptual frameworks, including the Institute of Medicine’s prevention taxonomy, which distinguishes between universal, selective, and indicated interventions (1). Universal prevention targets entire populations regardless of risk status, selective prevention focuses on individuals with elevated risk factors, and indicated prevention addresses those with early signs of mental health problems but not yet meeting diagnostic criteria (2). The socioecological model provides another fundamental theoretical framework, recognizing that mental health outcomes result from complex interactions between individual, interpersonal, community, and societal factors (3). This model emphasizes the importance of multilevel interventions that address risk and protective factors across different ecological levels, from individual psychological characteristics to broader social and policy environments (4).

Mental health, defined as a state of well-being encompassing emotional, psychological, and social dimensions (5), requires comprehensive preventive approaches rooted in health psychology principles (6). Contemporary conceptualizations of mental health prevention extend well beyond the reduction of diagnosable psychiatric disorders. The World Health Organization defines mental health promotion and prevention as a continuum encompassing the enhancement of positive wellbeing and resilience at the population level, as well as universal, selective, and indicated preventive interventions targeting populations of varying risk (7, 8). Within this framework, mental health promotion aims to strengthen protective factors and foster wellbeing irrespective of disorder risk, while preventive interventions—classified according to the Institute of Medicine taxonomy (1)—target the reduction of incidence, prevalence, and severity of mental disorders across risk strata. The present analysis operates primarily within the preventive intervention framework, focusing on universal, selective, and indicated strategies as operationalized in national policy documents and international comparative reports (8–10). This focus reflects the structure of available comparative data rather than a conceptual delimitation of the field; where evidence pertaining to mental health promotion is available and relevant, it is incorporated into the analysis.

Epidemiological evidence demonstrates the substantial burden of mental health disorders globally. In the European Union, mental health conditions affect approximately 84 million people (18.4% of the EU population of 447 million), representing substantial economic costs estimated at over €600 billion annually (9, 11, 32). Life-course epidemiology provides crucial insights into the timing and mechanisms of mental health prevention. Recent large-scale longitudinal studies confirm (12, 13) that approximately 75% of mental health disorders have their onset before age 24, with 50% beginning before age 14. This evidence supports the critical importance of early intervention and prevention programs targeting children and adolescents, when neuroplasticity and behavioral patterns remain most modifiable and intervention effects are most durable (14, 15, 33).

The prevention science literature (2, 16) has established robust evidence for the effectiveness of various preventive interventions. Meta-analytical reviews demonstrate that school-based prevention programs can reduce symptoms of depression and anxiety by 10%–15%, with effect sizes ranging from small to moderate (16). Social determinants of mental health theory emphasizes the fundamental role of structural factors in shaping population mental health outcomes. Recent meta-analytic evidence confirms that adverse social conditions, including poverty, discrimination, social isolation, and violence, significantly increase risk for mental health problems (17, 18). Conversely, protective social environments characterized by social cohesion, economic security, and access to quality education and healthcare promote population mental health (19, 34).

The implementation science framework provides essential guidance for translating evidence-based prevention programs into real-world settings. The Consolidated Framework for Implementation Research (CFIR) identifies key domains affecting implementation success, including intervention characteristics, outer setting factors, inner setting features, individual characteristics, and implementation processes (35). Understanding these factors is crucial for successful scaling of prevention programs across different healthcare and social service systems. Cross-national comparative research reveals substantial variation in mental health prevention approaches across European Union member states. Systematic reviews indicate that Nordic countries consistently demonstrate superior mental health outcomes and more comprehensive prevention systems compared to Central and Eastern European countries (19). These disparities reflect differences in healthcare system organization, resource allocation, political priorities, and cultural attitudes toward mental health (20).

The selection of Poland as the primary case for comparative analysis is justified on empirical, structural, and policy grounds. Poland constitutes the sixth-largest EU member state by population [approximately 38 million; (9)] and, as such, represents a health system of considerable demographic and epidemiological significance within the European region. From a structural perspective, Poland exemplifies a pattern documented across Central and Eastern European (CEE) post-transition health systems: the coexistence of formally developed legislative and strategic frameworks with persistent implementation deficits, including markedly low treatment coverage rates, below-average psychiatric workforce density, and insufficiently coordinated intersectoral provision (19, 20). This implementation gap renders Poland a theoretically productive case for examining the determinants of prevention system performance. Furthermore, the ongoing reform of child and adolescent psychiatric services—introduced through a phased, three-tier community care model (21)—provides a particularly timely policy context in which comparative evidence can directly inform reform trajectories.

The selection of comparator countries followed a purposive sampling strategy (22) aimed at maximizing variation in prevention system models while ensuring data comparability. Three country clusters were identified: (1) Nordic countries (Finland, Denmark, Sweden, Norway), representing systems with high mental health expenditure, well-developed universal prevention infrastructures, and strong intersectoral governance; (2) Western European countries (Netherlands, Luxembourg, Portugal), representing intermediate models with established but heterogeneous prevention systems; and (3) neighboring CEE countries (Czech Republic, Lithuania, Latvia), enabling within-region comparison and controlling for shared historical and structural factors. This selection is not intended to be exhaustive of all 27 EU member states; rather, it constitutes a theoretically grounded comparative basis aligned with the availability of systematic, internationally comparable data (9, 10, 23).

This study aims to analyze mental health prevention programs in Poland compared to selected European Union countries, examining both policy frameworks and implementation approaches. By applying theoretical frameworks from prevention science, implementation research, and comparative policy analysis, this research seeks to identify factors that facilitate or impede effective mental health prevention system development.

## 2 Methods

### 2.1 Study design

This comparative policy analysis employed a systematic document review methodology examining mental health prevention policies and programs across Poland and ten purposively selected EU member states from 2013–2025. The timeframe was selected to capture the period following the 2013 WHO Mental Health Action Plan and EU Joint Action on Mental Health and Wellbeing, which established contemporary frameworks for mental health prevention in Europe. The study follows principles outlined in guidance for policy document analysis in health systems research (22).

### 2.2 Data sources

We conducted systematic searches of multiple source types. The following databases were searched: Policy Databases: WHO Global Health Observatory and European Health Information Gateway; European Commission Public Health database; National government health ministry websites (Poland, Czech Republic, Finland, Denmark, Sweden, Norway, Netherlands, Lithuania, Latvia, Portugal, Luxembourg); OECD Health Statistics database; Eurostat database.

Academic Literature: PubMed/MEDLINE (2013–2025); Web of Science (2013–2025); Scopus (2013–2025). Full search strings used across academic databases: (“mental health prevention” OR “mental health promotion” OR “psychiatric prevention”) AND (“Poland” OR “Czech Republic” OR “Finland” OR “Denmark” OR “Sweden” OR “Norway” OR “Netherlands” OR “Lithuania” OR “Latvia” OR “Portugal” OR “Luxembourg” OR “European Union”) AND (“policy” OR “program” OR “intervention” OR “strategy” OR “national plan”). Searches were conducted in English only, without language restrictions on included documents. Search results were exported to a reference management system (Zotero) and deduplicated prior to screening. Grey Literature: National health strategy documents; Legislative acts and ministerial regulations; Reports from WHO Regional Office for Europe; European Commission reports; OECD mental health policy reviews. Selection Criteria.

Document selection followed a transparent, multi-stage screening process consistent with methodological guidance for policy document analysis in health systems research (22), described in full in Section 2.3.

### 2.3 Selection criteria

Documents were included if they: (1) addressed mental health prevention or promotion at national or EU policy levels, (2) were published between 2013–2025, (3) contained quantitative data on service provision or outcomes, and (4) described population-level, school-based, workplace-based, or community-based prevention approaches rather than individual interventions.

Exclusion criteria: (1) documents focusing solely on treatment rather than prevention, (2) individual facility-level reports without national relevance, (3) documents without verifiable authorship or publication source, (4) conference abstracts without full text availability. Documents were screened by two independent reviewers (DW, MB-M) first by title and abstract, then by full text. Discrepancies at each stage were resolved through discussion; unresolved discrepancies were adjudicated by a third researcher (AP). Inter-rater agreement at the full-text stage was calculated (Cohen’s *κ* = 0.82, indicating strong agreement). Total documents reviewed: 127 policy documents, 89 peer-reviewed articles, 34 statistical reports. Final inclusion: 42 policy documents, 28 peer-reviewed articles, 12 statistical reports.

### 2.4 Analysis framework

The analysis employed a comparative framework derived from two primary sources: the Institute of Medicine’s prevention classification system (1) and the WHO Mental Health Action Plan (8). Table 1 presents the analytical framework, showing each dimension, its theoretical basis, and the specific indicators extracted from the documents.

**Table 1:** Analytical framework for comparative policy analysis.

Analytical dimension	Theoretical basis	Indicators extracted
Universal prevention strategies	IOM taxonomy; WHO MHAP	Population-wide programs; school/workplace programs; anti-stigma campaigns
Selective interventions (at-risk populations)	IOM taxonomy	Programs targeting children, adolescents, low-SES groups, migrants
Indicated interventions (early signs)	IOM taxonomy	Early detection programs; crisis intervention services
Resource allocation and service accessibility	WHO MHAP; OECD benchmarks	Psychiatric workforce per 100,000; mental health budget % of health expenditure; treatment coverage rates
Legal and regulatory frameworks	Implementation science (CFIR)	National legislation; strategic plans; ministerial regulations
Intersectoral coordination mechanisms	Socioecological model	Education–health linkages; community partnerships; shared governance structures

IOM, institute of medicine; WHO MHAP, WHO mental health action plan 2013; CFIR, Consolidated framework for implementation research; SES, socioeconomic status.

Data extraction focused on: prevalence of mental health conditions, treatment coverage rates, workforce density (psychiatrists and nurses per 100,000 population), types of prevention programs implemented, target populations, policy implementation mechanisms, and funding allocations. Two researchers independently extracted data using a standardized template, with discrepancies resolved through discussion with a third researcher. Comparative analysis identified patterns, gaps, and best practices across countries, with particular attention to factors facilitating or impeding prevention program implementation.

## 3 Results

### 3.1 Mental health service accessibility in Poland

Poland demonstrates significant gaps in mental health service provision compared to EU standards. Among the 6 million individuals requiring psychiatric services, only 1.5 million (25%) receive adequate care (24). The healthcare workforce shows concerning deficits, with Poland providing 8.9 psychiatrists and 28.2 psychiatric nurses per 100,000 population, substantially lower than comparable economies such as Czech Republic (11.5 psychiatrists and 31.6 nurses per 100,000) and significantly below the EU average of 18 psychiatrists and 45 nurses per 100,000 population (25) (Table 2).

**Table 2:** Psychiatric workforce density in Poland and selected EU member states (25).

Country	Psychiatrists per 100,000	Psychiatric nurses per 100,000
Norway	30.3	n/a
Finland	22.7	55.4
Sweden	21.0	49.2
Netherlands	18.4	44.8
Denmark	17.9	48.1
EU average	18.0	45.0
Lithuania	14.2	36.7
Latvia	13.1	32.0
Portugal	12.7	34.5
Czech Republic	11.5	31.6
Luxembourg	10.8	29.4
Poland	8.9	28.2

n/a, not available. Sources: (23, 25).

The 2020 reform initiative by the Ministry of Health prioritized child and adolescent psychiatry through a three-tier care system: community psychological and psychotherapeutic centers at the primary level, community mental health centers with psychiatric consultation at the secondary level, and specialized 24-h psychiatric care facilities at the tertiary level (21).

### 3.2 Legal framework and policy implementation

Poland’s mental health prevention operates under several key regulatory frameworks: the Council of Ministers Regulation of March 30, 2021, regarding the National Health Program 2021–2025, and the Council of Ministers Regulation of February 8, 2017, concerning the National Mental Health Protection Program 2017–2022 (26, 27). The National Health Program specifically addresses mental health prevention and psychological wellbeing improvement, with suicide prevention and psychological crisis intervention across all age groups as priority areas.

Comparison with selected EU member states reveals that Poland’s policy architecture is broadly comparable in formal terms to many comparator countries—most have a national mental health strategy and legislative basis for mental health services. However, the critical distinction lies in implementation. Nordic countries (Finland, Denmark, Sweden) are characterized by dedicated ring-fenced mental health budgets, mandatory inter-ministerial coordination mechanisms, and statutory requirements for evidence-based program delivery in schools and workplaces. In contrast, Poland’s programs (Epsilon, Zippy’s Friends, Look Differently, ISKRA) remain largely voluntary, limited in reach, and concentrated on children below the age of 15, leaving a significant gap for older adolescents (aged 15–18). The Czech Republic presents a mid-range case: a policy architecture comparable to Poland’s but with somewhat greater workforce capacity and more systematic school-based mental health screening.

Despite policy frameworks, Poland lacks comprehensive systematic solutions. Schools lack unified support systems for children and parents, with fragmented coordination between education and health sectors. Existing evidence-based prevention programs (Epsilon, Zippy’s Friends, Look Differently) target children up to age 12, while the ISKRA resilience program addresses adolescents aged 11–15 (28), however its implementation remains limited and insufficient to meet population needs for older adolescents (aged 15–18).

### 3.3 EU member state prevention strategies

Analysis organized by prevention type (IOM taxonomy) reveals the following patterns. Universal prevention: Denmark, Finland, Netherlands, Lithuania, Latvia, Portugal, Luxembourg, and Sweden all report active population-wide suicide prevention programs. Finland, Latvia, and Portugal additionally implement national anti-stigmatization campaigns. Poland has not implemented a comparable systematic universal program; existing initiatives are project-based rather than institutionalized. Selective prevention: Nordic countries demonstrate the most developed selective programs, targeting children in low-income families, immigrant populations, and children with parents experiencing mental illness. Norway’s family intervention programs and Sweden’s school health services provide systematic early support. Poland’s selective programs are largely project-funded and time-limited, without a statutory framework ensuring continuity. Indicated prevention: Finland’s integrated approach combines early detection systems in schools with rapid referral pathways to community mental health services. Denmark has developed structured screening protocols for adolescents aged 12–18 in school settings. Poland does not have a national standardized screening protocol; school psychologists operate without a unified referral pathway.

In 2016, all EU countries reported progress in promotional and preventive activities (29). Nordic countries demonstrate particularly comprehensive approaches. Norway implemented a ten-year mental health promotion strategy, while Denmark developed school and workplace initiatives addressing suicide and marginalization (23). Sweden provided financial support to local authorities for mental health promotion, with the Swedish Public Health Agency designated to create national-level mental health promotion and disease prevention projects.

### 3.4 Resource allocation and service gaps

Comparative analysis reveals significant resource disparities. Mental health expenditure as a proportion of total health expenditure provides another indicator of system investment. Available OECD data indicate that Nordic countries allocate between 6 and 9% of total health expenditure to mental health, compared to an estimated 3%–4% in Poland (10). The EU benchmark target of 5%–7% recommended in European Commission guidance is met by most Nordic and Western European comparators but not by Poland or the Czech Republic. The fragmentation between sectors creates coordination challenges, with limited collaboration between educational institutions, healthcare providers, and social services. Universal prevention programs require substantial coordination across multiple sectors, which remains underdeveloped in Poland compared to Nordic and Western European models (9). The absence of systematic monitoring and evaluation frameworks further limits program effectiveness and scalability.

## 4 Discussion

This study aimed to analyze mental health prevention programs in Poland in comparison with selected EU member states, examining policy frameworks, resource allocation, and implementation approaches across universal, selective, and indicated prevention strategies. The findings reveal substantial and systematic disparities between Poland and comparator countries, particularly Nordic member states, across all analytical dimensions. These disparities are consistent with broader patterns documented in the European comparative literature, where post-transition Central and Eastern European health systems have consistently demonstrated lower prevention system performance relative to Western and Northern European counterparts (19, 20). The 25% treatment coverage rate in Poland—less than half the estimated EU norm—indicates profound unmet need at the population level (24), disproportionately affecting children, adolescents, and individuals from lower socioeconomic backgrounds, groups for whom early intervention carries the greatest long-term benefit (14).

The legal and policy infrastructure in Poland is formally comparable to many EU member states—national mental health programs exist and prevention is explicitly acknowledged as a priority (27). However, the present findings corroborate the observation of Kręglewska (30) and Szafrański (31) that formal policy commitment does not translate automatically into systematic implementation. This implementation gap is a well-documented phenomenon in health systems research and reflects structural factors including insufficient ring-fenced funding, fragmented governance across the education and health sectors, and an underdeveloped community mental health infrastructure. Critically, Poland’s existing school-based prevention programs (Epsilon, Zippy’s Friends, ISKRA) remain voluntary, project-funded, and concentrated among younger age groups, leaving adolescents aged 15–18 with no systematic preventive provision—a gap that is especially concerning given epidemiological evidence that the majority of mental health disorders emerge during precisely this developmental period (12, 13).

The workforce shortage in psychiatric services represents a critical barrier to prevention program implementation. With less than half the psychiatric workforce density (8.9 vs. 18 psychiatrists per 100,000) the workforce shortage in psychiatric services constitutes a structural impediment that underlies many of the prevention gaps identified in this analysis. With less than half the psychiatric workforce density of the EU average [8.9 vs. 18 psychiatrists per 100,000 population; (25)], Poland’s system is structurally oriented toward crisis management rather than prevention, a pattern consistent with findings from other under-resourced health systems (20). This resource constraint has a cascading effect: without adequate community-based mental health professionals, the three-tier reform model introduced in 2020 (21) faces significant implementation challenges at the primary and secondary care levels. In contrast, Nordic countries demonstrate that sustained investment in the mental health workforce, combined with clear institutional mandates for intersectoral coordination, enables the kind of proactive, prevention-oriented system architecture that Poland’s current reform aims to achieve. Finland’s integrated approach—combining population-level universal interventions, professional training in mental health literacy, and rapid community-based referral pathways—illustrates how structural alignment between policy commitment and resource allocation can translate into measurable population-level improvements (29). Sweden’s model of statutory financial support to local authorities for mental health promotion further demonstrates the importance of decentralized, adequately resourced implementation mechanisms (29). These comparative examples suggest that the critical determinant of prevention system performance is not the formal content of national policy documents, but the degree to which policy commitments are matched by ring-fenced funding, workforce capacity, and governance structures that enforce intersectoral coordination.

### 4.1 Study limitations and future development

The present findings should be interpreted in light of several methodological limitations. Cross-national data on mental health prevention programs vary substantially in definition, scope, and reliability, which constrains the precision of comparative inferences. The policy-level analytical focus does not capture sub-national or regional variation in implementation, which may be considerable, particularly in larger member states. The analysis covers the period 2013–2025, which encompasses significant structural disruptions including the COVID-19 pandemic; pandemic-related changes in service delivery and prevention program implementation may affect the generalizability of pre- and post-pandemic comparisons. Furthermore, the search strategy was conducted in English only, which may have resulted in underrepresentation of national-language policy documents and grey literature. Future research should incorporate native-language searches and longitudinal designs tracking population-level mental health outcomes following the implementation of specific prevention programs, particularly in the context of Poland’s ongoing psychiatric reform.

Future research should address several critical knowledge gaps. Longitudinal studies tracking population-level mental health indicators following prevention program implementation are essential for establishing causal relationships between policy interventions and outcomes. Randomized controlled trials and quasi-experimental evaluations of community-based prevention programs, particularly those targeting adolescents aged 13–18, would provide robust evidence for policy development. Comparative effectiveness research examining different governance models for intersectoral coordination could inform the design of prevention systems in countries currently undertaking reform.

## 5 Conclusion

This comparative analysis demonstrates that Poland’s mental health prevention system, while operating within a formally developed policy framework, faces substantial implementation challenges relative to selected EU member states. The findings suggest that bridging this gap will require coordinated progress across several interconnected domains, rather than isolated policy measures. Key areas for development include: (1) Workforce development: Progressively increase psychiatric workforce density toward EU benchmark levels through targeted recruitment, expanded training capacity, and improved professional conditions; (2) Systematic prevention programs: Establish universal mental health literacy programs in school settings, with particular attention to the currently underserved 15–18 age group, drawing on existing evidence-based models; (3) Intersectoral coordination: Develop formal governance mechanisms for collaboration between education, health, and social services, with defined accountability frameworks and shared performance indicators; (4) Resource allocation: Increase the proportion of health expenditure directed to mental health prevention, with targeted ring-fencing for prevention-specific programs, in line with WHO and OECD guidance; (5) Monitoring and evaluation: Invest in national data infrastructure to systematically track prevention program reach, implementation fidelity, and population-level outcomes over time.

Progress across these areas will require sustained political commitment, adequate and predictable funding, and engagement of multiple stakeholders across government, civil society, and the research community. The comparative evidence presented in this study indicates that the countries demonstrating the strongest prevention systems share a common feature: coherent alignment between policy goals, resource allocation, and implementation governance. Poland’s ongoing psychiatric reform provides a timely opportunity to incorporate these lessons while developing solutions appropriate to its specific institutional and cultural context.

## Data Availability

The original contributions presented in the study are included in the article/supplementary material, further inquiries can be directed to the corresponding author.
